# Eosinophilic Liver Abscess with Charcot-Leyden Crystals

**DOI:** 10.4269/ajtmh.24-0745

**Published:** 2025-04-01

**Authors:** Sanjeev Sachdeva, Ashok Dalal, Surbhi Goyal

**Affiliations:** ^1^ Department of Gastroenterology, GB Pant Hospital, New Delhi, India;; ^2^ Department of Pathology, GB Pant Hospital, New Delhi, India

**Figure 1.**
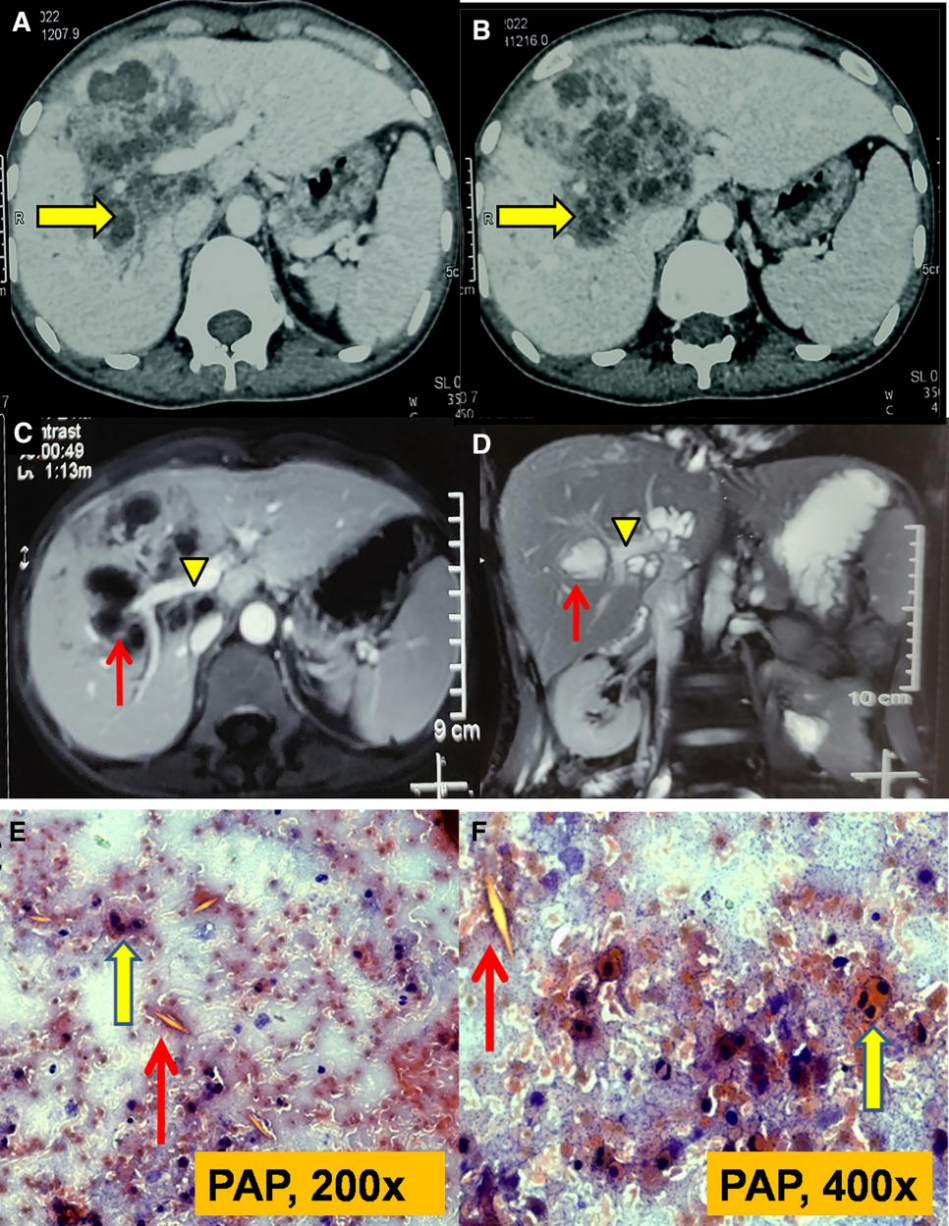
(**A** and** B**) Computed tomography (CT) of abdomen showing multiple conglomerated hepatic abscesses (arrows) involving segments 3, 5, and 8. (**C** and **D**) Triphasic magnetic resonance imaging (MRI) of abdomen showing multiseptated lesion with internal areas of necrosis (arrows) in right lobe of liver in close proximity with right branch of portal vein (arrowheads); and (**E** and **F**) Ultrasound-guided fine needle aspiration cytology (FNAC) from the hepatic lesion showing multiple eosinophilic infiltrates (thick arrows) with Charcot-Leyden crystals (arrows) consistent with parasitic infection (PAP stain [200×, 400×]). PAP = papanicolaou.

**Figure 2.**
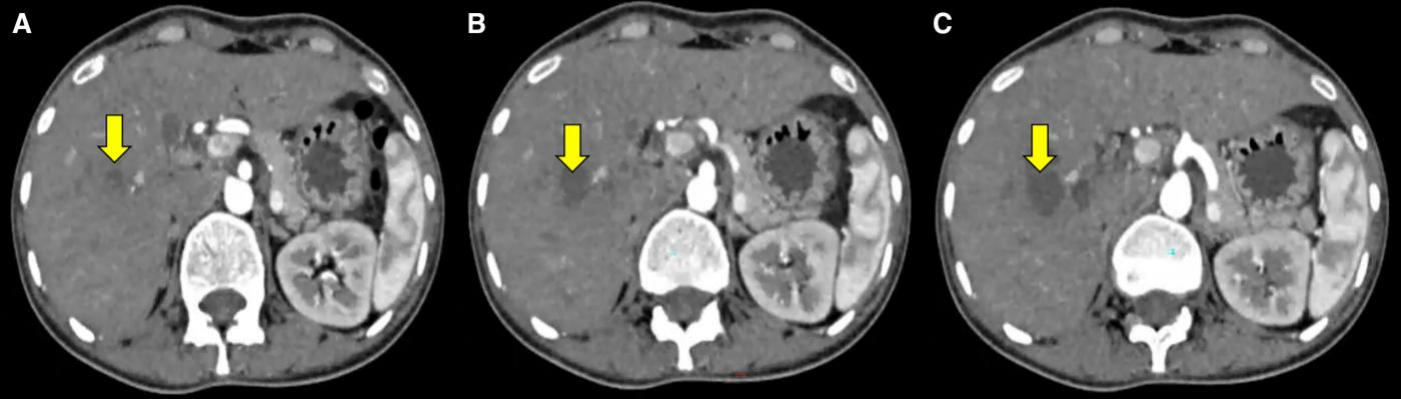
(**A–C**) Computed tomography (CT) of abdomen at 6 weeks posttreatment showing remarkable improvement with resolving small lesions in right lobe of liver (arrows).

A 42-year-old female presented with right upper-quadrant abdominal pain, low-grade fever, anorexia, and significant weight loss for 6 months. Examination revealed mild pallor and hepatomegaly. Her hemoglobin was 10.3% gm, eosinophil fraction of differential leukocyte count was 25% with absolute eosinophil count of 1,860/cumm, but liver function tests were normal. Ultrasound abdomen showed hepatomegaly with multiple conglomerated abscesses in the right lobe of liver. Computerized tomography of abdomen showed multiple conglomerated hepatic abscesses (arrows) involving segments 3, 5, and 8 (Figure 1A and B). Triphasic magnetic resonance imaging of abdomen revealed multiseptated lesion with internal areas of necrosis (arrows) in right lobe of liver in close proximity with right branch of portal vein (arrowheads) (Figure 1C and D). Ultrasound-guided fine needle aspiration cytology (FNAC) from the hepatic lesion revealed multiple eosinophilic infiltrates (thick arrows) with Charcot-Leyden crystals (arrows) consistent with parasitic infection (Figure 1E and F). Stool microscopic examination was normal on 3 consecutive days. Her *Toxocara* serology (IgG) by ELISA (NovaLisa^®^ [Gold Standard Diagnostics, Dietzenbach, Germany]) was positive but hydatid serology (IgG) by ELISA (demeditec [Demeditec Diagnostics GmbH, Kiel, Germany]) was negative. She was given oral albendazole therapy, subsequent to which her symptoms started improving within 4 weeks. There was more than 80% resolution of the lesions at follow-up imaging at 6 weeks (Figure 2). Because our case serology for *Toxocara* was positive and response to treatment was good, it was likely toxocariasis; however, direct visualization of the organism would have been substantially more supportive.

Liver is a common site of involvement in several parasitic infections. Human hepatic toxocariasis is a rare parasitic disease. *Toxocara* related visceral larva migrans^1^^,^^2^ may manifest as eosinophilic liver abscess rich in Charcot-Leyden crystals.^3^^,^^4^ Differential diagnosis includes other hepatic parasitic infections. First line of management of this unusual condition is albendazole therapy.^5^
